# Kinetics of Leptin Binding to the Q223R Leptin Receptor

**DOI:** 10.1371/journal.pone.0094843

**Published:** 2014-04-17

**Authors:** Hans Verkerke, Caitlin Naylor, Lennart Zabeau, Jan Tavernier, William A. Petri, Chelsea Marie

**Affiliations:** 1 Department of Medicine, Division of Infectious Diseases and International Health, University of Virginia, Charlottesville, Virginia, United States of America; 2 Flanders Institute for Biotechnology (VIB), Department of Medical Protein Research, Ghent University, Ghent, Belgium; Institute of Enzymology of the Hungarian Academy of Science, Hungary

## Abstract

Studies in human populations and mouse models of disease have linked the common leptin receptor Q223R mutation to obesity, multiple forms of cancer, adverse drug reactions, and susceptibility to enteric and respiratory infections. Contradictory results cast doubt on the phenotypic consequences of this variant. We set out to determine whether the Q223R substitution affects leptin binding kinetics using surface plasmon resonance (SPR), a technique that allows sensitive real-time monitoring of protein-protein interactions. We measured the binding and dissociation rate constants for leptin to the extracellular domain of WT and Q223R murine leptin receptors expressed as Fc-fusion proteins and found that the mutant receptor does not significantly differ in kinetics of leptin binding from the WT leptin receptor. (WT: k_a_ 1.76×10^6^±0.193×10^6^ M^−1^ s^−1^, k_d_ 1.21×10^−4^±0.707×10^−4^ s^−1^, K_D_ 6.47×10^−11^±3.30×10^−11^ M; Q223R: k_a_ 1.75×10^6^±0.0245×10^6^ M^−1^ s^−1^, k_d_ 1.47×10^−4^±0.0505×10^−4^ s^−1^, K_D_ 8.43×10^−11^±0.407×10^−11^ M). Our results support earlier findings that differences in affinity and kinetics of leptin binding are unlikely to explain mechanistically the phenotypes that have been linked to this common genetic variant. Future studies will seek to elucidate the mechanism by which this mutation influences susceptibility to metabolic, infectious, and malignant pathologies.

## Introduction

Leptin is a secreted adipocytokine that regulates energy expenditure. Centrally, leptin acts on hypothalamic circuits to inhibit food intake. Peripherally, it stimulates immune and barrier cells to promote activation, proliferation, resistance to cell death, and wound repair [1]. Leptin is known to engage the cytokine receptor homology-2 (CRH2) domain in the extracellular region of the leptin receptor (LepR) with high affinity [2]–[3]. Upon leptin binding and clustering of activated receptor complexes, Janus Kinase 2 (JAK2) phosphorylates intracellular tyrosine residues, which recruit transcription factors: Src Homology Phosphatase 2 (SHP2), Signal Transduscer and Activator of Transcription 5 (STAT5), and STAT3. SHP2 positively regulates the Extracellular Signal-Regulated Kinase/c-fos (ERK/c-fos) pathway. Activated and dimerized STAT3 translocates to the nucleus where it initiates a transcriptional program, which includes the upregulation of SOCS3, a feedback inhibitor of leptin signaling [4].

The Q223R encoding SNP (rs1137101) is exceedingly common and widely distributed. According to data from the 1000 genomes project, the overall allelic frequency is 41% A, encoding glutamine, and 59% G, encoding arginine. Interestingly, frequencies vary significantly by region and ethnicity [5]. Numerous studies have found modest or strong association of this variant with obesity and adiposity [6], multiple forms of cancer [7]–[9], peritonitis [10], adverse drug reactions [11] and susceptibility to enteric [12]–[15] and respiratory [16] infections after controlling for ethnicity, age, sex, and environmental factors. However, studies in separate populations have found no significant association for several of these conditions [17]–[18]. In 2011, a meta-analysis of published findings on the Q223R variant suggested that heterogeneity in association between this mutation and body weight in human populations may be attributable to differences in study design and power [19].

LepR contains two extracellular CRH domains separated by an immunoglobulin-like domain. Two fibronectin type 3 (FN III) domains separate the CRH2 domain from a single transmembrane region and a long intracellular signaling region containing a membrane-proximal box 1 motif [2]. The LepR Q223R mutation results from an A to G transversion encoding a glutamine to arginine substitution in the N-terminal CRH1 domain [20]. It has been shown that the CRH2 domain is both necessary and sufficient for leptin binding; while CRH1 is dispensable for a high affinity interaction [21].

To test the hypothesis that the Q223R amino acid replacement affects ligand-binding kinetics of the leptin receptor, we measured the leptin/LepR interaction using SPR technology on a BiaCore T200 platform. To measure leptin association and dissociation for both the mutant and wild type receptors, the extracellular domain of each receptor was expressed as an Fc-fused chimera and immobilized to a dextran matrix coated with anti-IgG antibody. Increasing concentrations of leptin were then injected into the system. The rates of binding and dissociation were monitored at each leptin concentration in real-time as changes in the refractory index of the capture surface measured in response units (RUs). The ability to monitor these interactions in real-time allowed for the determination of binding kinetic constants by global fitting to a model of 1∶1 ligand to receptor binding.

## Materials and Methods

### Tissue Culture, Transfection, and Concentration

HEK293T/17 cells were maintained in a humidified incubator at 37°C with 5% CO_2_ and cultured in HEPES buffered Dulbecco's Modified Eagle's Medium-F12 (Gibco) supplemented with 10% heat-inactivated fetal bovine serum (FBS). pMET7 constructs encoding the WT or Q223R extracellular domain of the murine LepR, C-terminally fused to the Fc region of murine IgG1, a FLAG-tag, and a 6x His tag were obtained from the Cytokine Receptor Lab at Ghent University. Sequences of the WT and Q223R constructs were confirmed by Sanger sequencing with T7 primers provided by Genewiz, inc. When HEK293T/17 cells were between 70 and 90% confluent, transfection using Lipofectamine 2000 (Invitrogen) was performed per the product instructions using WT, Q223R, or empty vector plasmid DNA. 12 hours after transfection, monolayers were washed and growth medium was replaced with serum free Optimem (Gibco) supplemented with 2 mM sodium butyrate. 48 to 72 hours later, supernatants were harvested and cleared of cellular debris by centrifugation.

Cleared supernatants were concentrated 20x in Amicon Ultra 15 ml centrifugal filter devices with a nominal molecular weight limit of 100 kDa at 3000×*g* for 15 minutes. Buffer exchange to 1x PBS was performed following the instructions of the filtration device manufacturers (Millipore) before BiaCore analysis.

### Immunoblotting

Supernatants were heated to 95°C in 4 x SDS-PAGE sample buffer for 5 minutes and separated by molecular weight using SDS-PAGE in Mini-PROTEAN TGX Precast Gels (4–20%). Replicate gels were either stained using Coomassie blue or proteins were transferred to polyvinylidene difluoride (PVDF) membranes by standard wet transfer methods. PVDF membranes were blocked with 5% milk in tris-buffered saline−0.05% Tween 20 (TBST) for 1 hr. Blots were either probed in two steps using anti-murine LepR (R&D) and a horseradish peroxidase (HRP)-conjugated secondary antibody (Sigma Aldrich) or in one step using HRP-conjugated anti-mouse IgG1 (Sigma Aldrich). Ab specific HRP-conjugated anti-mouse IgG1 was used as a control for non-specific binding to residual IgG components in the expression medium. Blots were washed 3 times for 5 minutes in TBST between probing steps and antibody bound proteins were visualized with ECL reagents (Pierce). Stained gels were imaged using a typhoon fluorescent imager (GE).

### Surface Plasmon Resonance

The BiaCore biosensor T200, CM5 biosensor chips, N-hydroxysuccinimide (NHS), N-ethyl-N-(3-diethylaminopropyl)carbodimid (EDC), ethanolamine-HCl, and HBS-EP buffer were obtained from BiaCore AB (GE). Rabbit polyclonal anti-mouse IgG1 was purchased from GE Healthcare. Recombinant murine leptin (MW 16 kDa) was obtained from R&D Systems Europe, Ltd. Anti-mouse IgG1 was immobilized on two channels of a carboxymethyl dextran chip (CM5) by amine coupling to the dextran matrix, activated with NHS/EDC for 5 minutes. The antibody was injected at 200 µg/ml in sodium acetate buffer (10 mM, pH 4.0). Approximately 2000 resonance units (RU) of antibody were coupled in each channel for each experiment. Blocking of the activated surface was achieved with a 5 minute injection of 1 M ethanolamine (pH 8.5).

A typical run at 25°C involved the injection of 100-200 µl of Amicon-concentrated extracellular murine leptin receptor Fc (LepR_ec_-Fc) chimera to achieve 100–300 RU of immobilized receptor. The surface was equilibrated for 6 minutes prior to leptin or HBS-EP buffer injection. A range of leptin concentrations (1.25–60 nM) was tested for each receptor chimera and their association was monitored for 600 seconds. The dissociation phase was monitored for 1000 seconds before regeneration of the surface using 10 mM glycine, pH 2.0. The flow rate was set to 30 µl/min. To monitor nonspecific binding, an antibody-coated channel lacking bound LepR_ec_-Fc was run with leptin and HBS-EP buffer. Sensorgrams were generated from reference-subtracted leptin binding data for each receptor.

### Statistics and Data Analysis

Kinetic parameters were derived using BIA evaluation software 3.1 (BiaCore AB). A non-linear least squares analysis model for 1∶1 binding was applied to fit data from association and dissociation phases simultaneously and globally across all leptin concentrations tested. Means and SEM were calculated from two separate experiments at the indicated concentrations of leptin. A student's t-test was performed on rates of association and dissociation for each receptor.

## Results

### Expression and concentration of LepR_ec_-Fc chimeras

**Figure 1:**
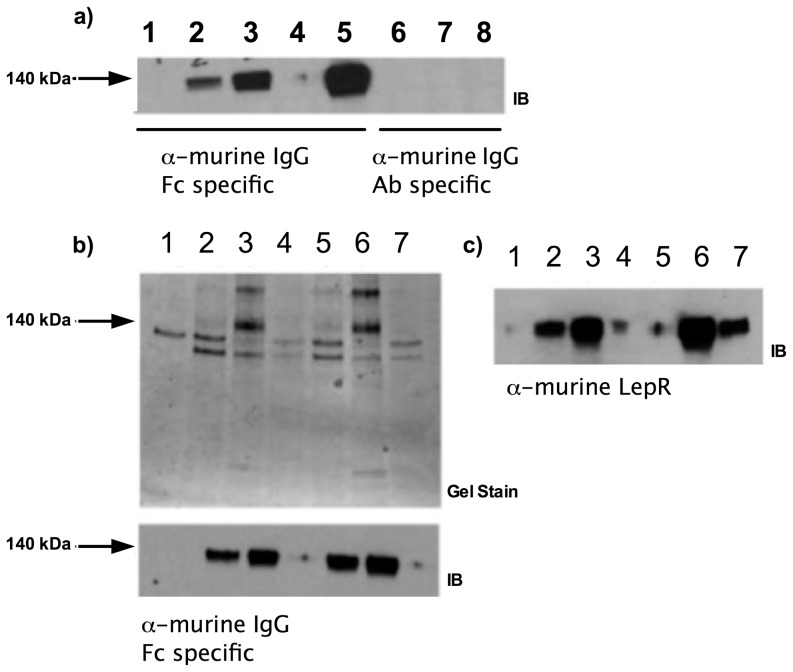
Expression and isolation of recombinant murine LepR_ec_-Fc fusion proteins. **a**) Expression of murine LepR_ec_-Fc constructs in adherent HEK293T/17 cells. Supernatants containing LepR_ec_-Fc chimera proteins from 24 and 48 hours post-transfection were cleared of cellular debris, subjected to SDS PAGE and western blotted with antibodies against domains of murine IgG1 (Fc specific for lanes 1–5 and Ab specific for lanes 6–8). **Lane 1**: Mock transfected at 48 hours. **Lane 2**: WT transfected at 24 hours. **Lane 3**: WT transfected at 48 hours. **Lane 4**: Q223R transfected at 24 hours. **Lane 5**: Q223R transfected at 48 hours. **Lane 6**: Mock transfected at 48 hours. **Lane 7**: WT transfected at 48 hours. **Lane 8**: Q223R transfected at 48 hours. **b**) Concentration and buffer exchange of murine LepR_ec_-Fc chimeras. Supernatants were collected 48 hours after growth medium was replaced with expression medium (serum free Optimem +2 mM sodium butyrate). These supernatant preparations were concentrated by Amicon ultrafiltration (NMWCO of 100 kDa). Supernatants, concentrates, and filtrates from each chimera were subjected to SDS-PAGE followed by coomassie staining (top) and western blotting with α-murine IgG1 specific to the Fc region (bottom). **Lane 1**: Expression medium. **Lane 2**: WT supernatant. **Lane 3**: WT concentrate. **Lane 4**: WT filtrate. **Lane 5**: Q223R supernatant. **Lane 6**: Q223R Concentrate. **Lane 7**: Q223R Filtrate. **c**) Supernatants, concentrates, and filtrates (as in b) from each chimera were subjected to SDS-PAGE followed by transfer to PVDF membrane and western blotting with α-murine leptin receptor (R&D scientific). **Lane 1**: Expression medium. **Lane 2**: WT supernatant. **Lane 3**: WT concentrate. **Lane 4**: WT filtrate. **Lane 5**: Q223R Filtrate. **Lane 6**: Q223R Concentrate. **Lane 7**: Q223R supernatant.

Q223R and WT murine LepR_ec_-Fc chimeras were expressed in the supernatants of adherent HEK293T/17 cells. Each preparation was concentrated by ultrafiltration with a nominal molecular mass limit of 100 kDa. Because concentrated preparations used in BiaCore experiments were not affinity purified, we modified the BiaCore immobilization step to use anti-murine IgG1 as the capture molecule instead of direct coupling. Murine leptin (the analyte) was obtained from R&D and resuspended in phosphate buffered saline at 1 mg/ml before dilution in BiaCore running buffer to the appropriate concentrations. **Figure 1** shows analysis of expression (a) and concentration steps (b,c) in the preparation of the LepR_ec_-Fc chimeras.

### Binding kinetic analysis of WT and Q223R LepR_ec_-Fc with recombinant leptin

**Figure 2:**
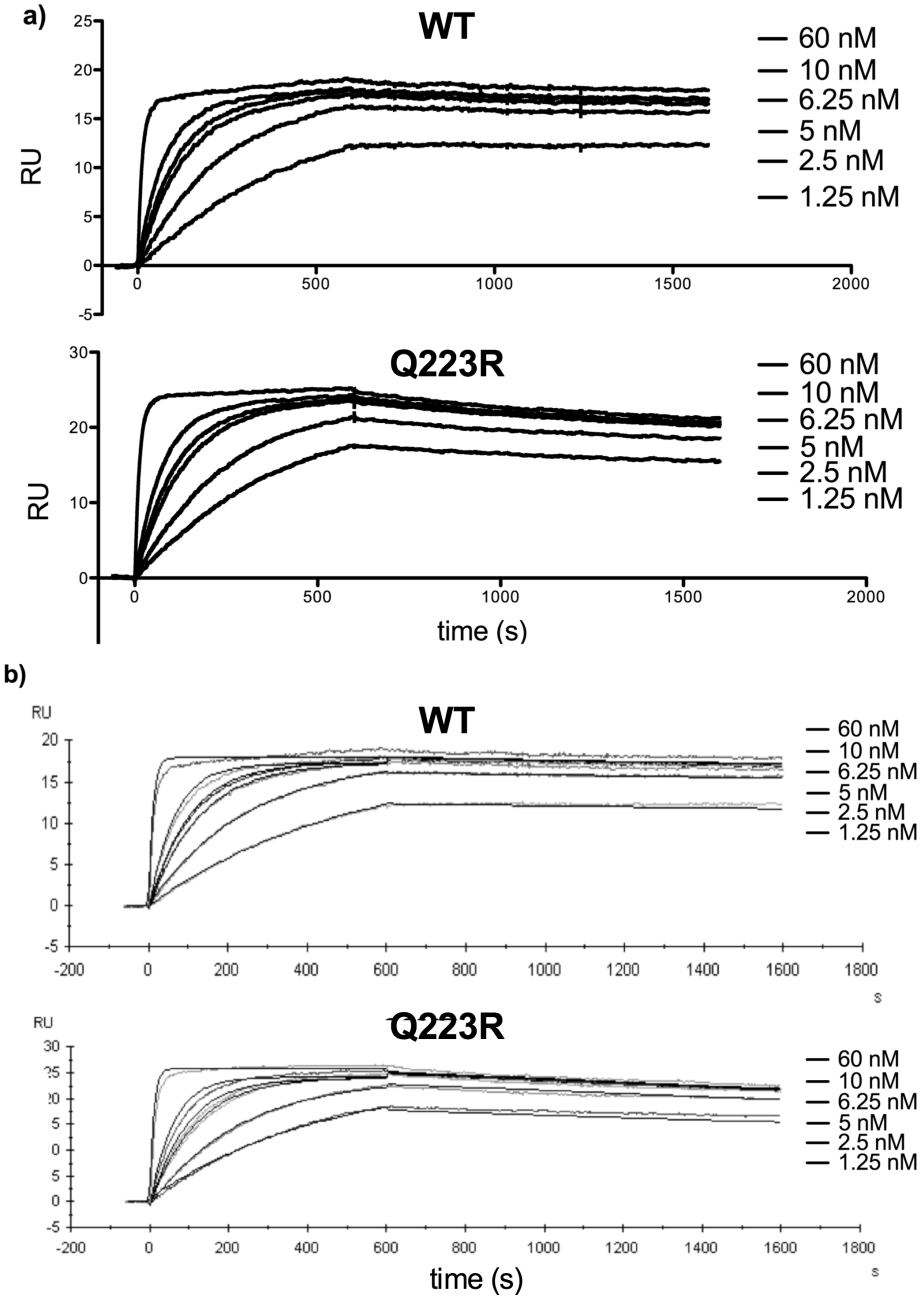
Surface plasmon resonance kinetic analysis of leptin binding to Q223R and WT murine LepR_ec_-Fc fusion proteins. **a**) Sensorgrams from SPR experiments using Amicon-concentrated WT and Q223R murine LepR_ec_-Fc chimeras and recombinant murine leptin at concentrations from 1.25 to 60 nM. After receptor immobilization on a chip coated with Fc specific α-murine IgG1, each concentration of leptin was injected for an association time of 600 seconds followed by 1000 seconds of monitoring ligand dissociation. **b**) Following background subtraction, kinetic parameters were derived from global analysis of sensorgrams based on a model of Langmuir (1∶1) binding kinetics using BiaCore T200 software for both WT and Q223R sensorgrams. Fitted curves (thin black lines) are overlaid on sensorgrams to demonstrate fitting to the binding model.

Kinetic parameters were derived from global Langmuir fitting for 1∶1 binding kinetics to sensorgrams of leptin-LepR binding at 1.25, 2.5, 5, 6.25, 10, and 60 nM murine leptin. Values for the on rate (k_a_), off rate (k_d_), and dissociation constant (K_D_) were derived from global fits from two separate experiments using independently prepared protein for the WT and Q223R chimera receptors. **Figure 2** shows representative sensorgrams depicting leptin binding and dissociation with reference surface background subtracted alone (**Figure 2a**) and overlaid with globally fitted curves to the 1∶1 model of leptin binding (**Figure 2b**) for WT **and** Q223R. Binding and dissociation are shown in response units (RU). Because the preparations containing the WT receptor contained less protein than the Q223R preparations, less of the WT chimera was immobilized before leptin binding and a lower maximum concentration was bound during the kinetic runs. However, sufficient receptor was immobilized to derive kinetic constants for each run and differences of this magnitude are unlikely to alter kinetic determinations.

Two subnanomolar affinity interactions were detected and monitored in our system to derive kinetic constants for each receptor. Standard errors were calculated and student's t-tests performed on data from two separate experiments for the three parameters. Derived constants are summarized in **Table 1**. No significant difference was measured and we concluded that the Q223R amino acid replacement does not significantly alter 1∶1 binding kinetics of murine leptin binding to the murine leptin receptor.

**Table 1:** Association and dissociation kinetics of Q223R and WT LepR_ec_-Fc fusions.

LepR type	k_a_ (10^6^ M^−1^ s^−1^)	k_d_ (10^−4^ s^−1^)	K_D_ (10^−11^ M)	χ^2^
**WT**	1.76±0.193	1.21±0.707	6.47±3.30	0.13–0.151
**Q223R**	1.75±0.0245	1.47±0.0505	8.43±0.407	0.26–0.35
**P value**	0.931	0.744	0.615	______

Kinetic constants derived from two independent binding affinity studies for each receptor (mean ± standard error). Association (k_a_) and dissociation constants (k_d_) were measured in real time using surface plasmon resonance for the WT and Q223R extracellular murine LepR-mFc fusion chimeras. P-values were determined using a student's t-test and data from two kinetic runs for each receptor. A p-value of less than 0.05 would have been considered significant. The range of **χ^2^** values is included to assess closeness of fit to the model of 1∶1 binding used in our analyses. **χ^2^**<2 is indicative of acceptable fit to the model.

## Discussion

The most important finding of this work is that the common Q223R encoding SNP in the LepR extracellular domain does not affect the rates at which leptin binds to and dissociates from its receptor. We investigated whether the Q223R substitution could alter ligand binding using surface plasmon resonance (SPR). Because it is both common and non-conservative, the Q223R amino acid replacement has been among the most studied variants of LepR. However, the functional consequences of this variant remain poorly defined. This mutation occurs in the CRH1 domain, which is dispensable for high affinity leptin binding, but may have roles in downstream signaling, receptor trafficking, or surface expression [21]–[23].

Stratigopoulos et al. observed no difference in adiposity between WT and Q223R isogenic mice fed a high fat diet [20]. A recent study found no significant association between this LepR polymorphism and obesity in humans. However, the homozygous Q223R encoding genotype did correlate strongly with increased serum cholesterol and low density lipoprotein (LDL) in both obese and non-obese subjects [24]. In addition, our earlier work has demonstrated a dramatic association between the human Q223R LepR mutation and amebiasis, a common cause of diarrheal morbidity and mortality among children globally [12]. Furthermore, patients carrying the Q223R mutation are at increased risk of *Clostridium difficile* colitis [15].

SPR was used to measure, in real-time, the interaction between murine leptin and the extracellular domain of murine LepR with or without the Q223R mutation. Until recently, the two primary models of leptin binding have suggested leptin:LepR stoichiometries of 2∶2 or 2∶4 [25]–[26]. However, a single particle electron microscopy study by Mancour et al. provided strong evidence for a ligand-induced native 2∶2 quaternary structure for leptin and its receptor [2]. We sought to address our primary question in a minimalist fashion, focusing on the interaction between one extracellular domain of the leptin receptor and one leptin molecule. Future studies will seek to investigate the role of the Q223R mutation in oligomerization of the extracellular domain, which could explain mechanistically attenuated leptin signaling observed in cells transfected with this variant [14].

Using SPR, Mistrik et al. measured values similar, but not identical, to our own for k_a_, k_d_, and K_D_ with murine leptin and murine extracellulary LepR bivalently fused to an Fc domain (1.5×10^6^ M^−1^s^−1^, 7×10^6^ s^−1^, and 0.5 nM respectively) [26]. Alternative approaches using radioligand binding have also yielded dissociation constants in the sub-nanomolar range [27]–[28]. However, Mancour et al. measured a markedly different K_D_ between murine leptin and the extracellular domain of murine LepR (17 nM) using isothermal titration calorimetry [2]. Studies specifically comparing SPR to ITC have yielded comparable dissociation constants for multiple molecular interactions [29]. However, a systemic comparison of techniques will be required to satisfactorily address discrepancies in parameters derived by these two technologies for the leptin receptor.

One limitation of SPR is the challenge of modeling molecular interactions with low dissociation rates. The difference between the K_D_ value that we measure and that determined using SPR by Mistrik et al. is due largely to differences in measured dissociation rates (k_d_). Even after 1000 seconds of monitoring in our system, only a fraction of bound ligand had dissociated from both mutant and WT receptor. This observation could in part be due to rebinding of leptin during the dissociation phase, a common confounding variable in SPR. It is also possible that a small contribution of mass transport in our study complicated accurate derivation of kinetic constants. Despite these limitations, the key finding of our work was internally consistent and indeed supports the conclusion that no significant difference in binding kinetics exists between Q223R and WT LepR.

It remains possible that this mutation alters cell-signaling networks by affecting surface expression, oligomerization kinetics, or receptor turnover. In fact, we have shown previously using a STAT3 driven luciferase reporter assay that cells transfected with the Q223R receptor exhibit lower signaling via STAT3 relative to cells expressing WT LepR [14]. Furthermore, Zabeau et al., showed that deletion of the entire CRH1 domain altered optimal downstream signaling via JAK2 [23]. The current study demonstrates clearly that these signaling phenotypes are unlikely to arise from kinetics of receptor-ligand binding and represents a step toward understanding how a mutation in the distal CRH1 domain of the leptin receptor affects human health and disease.
